# Clinical validation of the Tempus xO assay

**DOI:** 10.18632/oncotarget.25381

**Published:** 2018-05-25

**Authors:** Nike Beaubier, Robert Tell, Robert Huether, Martin Bontrager, Stephen Bush, Jerod Parsons, Kaanan Shah, Tim Baker, Gene Selkov, Tim Taxter, Amber Thomas, Sam Bettis, Aly Khan, Denise Lau, Christina Lee, Matthew Barber, Marcin Cieslik, Casey Frankenberger, Amy Franzen, Ali Weiner, Gary Palmer, Robert Lonigro, Dan Robinson, Yi-Mi Wu, Xuhong Cao, Eric Lefkofsky, Arul Chinnaiyan, Kevin P. White

**Affiliations:** ^1^ Tempus Labs, Inc., Chicago, Illinois 60654, USA; ^2^ Department of Pathology and Michigan Center for Translational Pathology, University of Michigan, Ann Arbor, Michigan 48109, USA; ^3^ Howard Hughes Medical Institute, Chevy Chase, Maryland 20815, USA

**Keywords:** genomic test, gene panel, cancer, transcriptome, Tempus

## Abstract

We have developed a clinically validated NGS assay that includes tumor, germline and RNA sequencing. We apply this assay to clinical specimens and cell lines, and we demonstrate a clinical sensitivity of 98.4% and positive predictive value of 100% for the clinically actionable variants measured by the assay. We also demonstrate highly accurate copy number measurements and gene rearrangement identification.

## INTRODUCTION

Clinically validated next-generation sequencing (NGS) assays are fundamental to fulfilling the promise of improved and individually targeted therapies that precision medicine holds for cancer patients. Comprehensive genomic profiling using deep, high-quality, clinically certified (CAP/CLIA) laboratory sequencing has emerged as a key tool in clinical decision support [1, 2]. Since the initial draft of the human reference genome was released in 2000 [3, 4], an extraordinary amount of genomic data has been collected. Comprehensive NGS based scans of cancer genomes were first published in 2007 [5], and for the past decade a great deal of scientific energy has focused on the hunt for oncogenes and tumor suppressor genes that drive cancers [6]. The concomitant growth of targeted therapies has led to the development of new clinical assessment strategies that routinely incorporate NGS to detect targetable mutations or mutational signatures, thus leading to improved survival and increased quality of life for a growing number of patients [7].

With the advent of NGS it has become possible to accurately detect genetic alterations in relevant cancer genes in a single comprehensive assay with high sensitivity and specificity. This has cost advantages as well as providing better stewardship of limited biopsy material. However, the use of this technology in a clinical context as a routine test faces numerous challenges. Firstly, nearly all clinical specimens are formalin fixed paraffin embedded tissue (FFPET), which can have degraded DNA and RNA. Thus, robust nucleic acid extraction protocols and sequencing library construction protocols must be applied. Secondly, many samples available for testing contain limited amounts of tissue, which in turn limits the amount of nucleic acid it is possible to obtain. Thirdly, accurate profiling in clinical specimens requires a sensitive enough assay to detect gene alterations in specimens with a low tumor percentage. Therefore, there must be deep coverage across all targeted regions and appropriately designed analysis algorithms. Lastly, because millions of bases within the tumor genome are assayed, rigorous statistical and analytical approaches for validation are required in order to demonstrate the accuracy of NGS technology for use in the clinical setting.

Here we present a validated, NGS-based oncology assay that interrogates 1,711 cancer-related genes (Supplementary Table 1) in matched tumor and normal tissue with whole transcriptome RNA sequencing for gene rearrangement detection. We refer to this cancer genome profiling test as the Tempus xO assay. We have instituted performance benchmarks supporting clinical use of Tempus xO and have assessed analytical sensitivity, specificity, accuracy and precision across the test's reportable range. The Tempus xO assay was validated with tumor DNA and RNA isolated from FFPET and germline DNA isolated from blood or saliva. Base substitutions, insertions and deletions (indels), focal gene amplifications and homozygous gene deletions of tumor and germline were assayed through DNA hybrid capture sequencing. Gene rearrangement events were assayed through RNA sequencing. The Tempus xO gene panel has been designed to specifically target therapeutically actionable genes, referred to as Tier 1 genes (Supplementary Table 2), defined as genes linked with response or resistance to targeted therapies, resistance to standard of care, or toxicities associated with treatment based on an extensive literature review. Literature review included evidence from clinical trials, clinical research, case studies and pre-clinical studies, and was paired with information about specific variants from the Catalogue of Somatic Mutations in Cancer (COSMIC). Furthermore, the Tempus xO assay allows for clinical trial routing based on the most recent literature and clinical trial availability.

## RESULTS

### *In silico* algorithm performance: PPV and sensitivity

The synthetic tumor samples had consistently had 0 false positives at 25% tumor titers. In the 15% sample, there was 1 false positive detected, and in the 5% sample there were 0 false positives detected of the 110 total variants. These findings result in an algorithmic PPV of 1.00, 0.99, and 1.00, respectively. Subsequent to PPV analysis, samples were analyzed for algorithmic sensitivity. In the 25% synthetic tumor titers, there was consistently 1 false negative variant left out by the pipeline. In the 15% sample, there was 1 false negative variant missed by the pipeline, and in the 5% sample, 6 false negatives were missed of the 110 variants. These findings result in an algorithmic sensitivity of 0.99, 0.99, and 0.93, respectively.

### Experimental concordance to three reference laboratories: assay sensitivity and specificity

**Figure 1:**
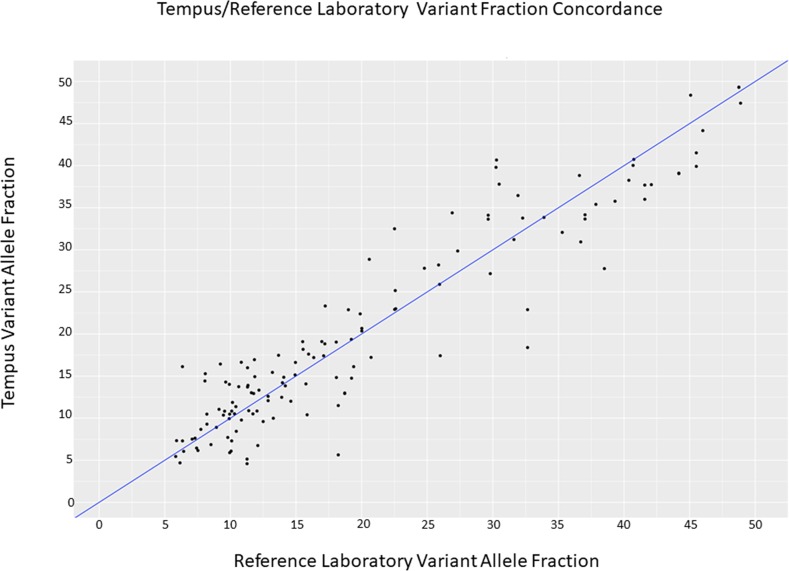
Analytical concordance between Tempus Labs and MCTP somatic variant analysis Reported Variant Allele Fractions from 18 representative tumor-normal pairs were compared for variants detected at Tempus and MCTP, representing a range of different sample types and sample qualities. Across a range of variant fractions commonly present within clinical cancer samples we observed an r-squared value of 0.884. Data are distributed around the line where x=y (blue).

In order to analyze sensitivity, 19 clinical cancer samples were sent to Neogenomics Inc., a molecular pathology laboratory that utilizes a clinical 48 gene targeted oncology panel and 22 samples were tested at Northwestern Memorial Hospital (NMH). The NMH samples had been tested for amplification using diagnostic standard of care (fluorescence in situ hybridization assay) and were used for determining sensitivity and specificity for calling of copy number amplifications. Furthermore, the NMH samples were sent to a partner reference laboratory at MCTP in order to be sequenced at high depth with the same 1711 gene xO panel used at Tempus. Figure 1 shows a Pearson correlation of 0.884 for VAFs across the 145 concordant variants tested between Tempus and MCTP (see Supplementary Figure 2 for additional coefficient of variation analysis of VAFs).

Utilizing this methodology, critical performance parameters were measured in terms of absolute sensitivity and absolute specificity within the context of single nucleotide variants (SNVs), small indels, and copy number variation.

### Assay sensitivity

The Neogenomics assay is known to be of high sensitivity and specificity for clinically relevant targeted genes, and it was thus used as a comparator for the Tempus xO sensitivity study. Samples without associated metadata were sent to Neogenomics while being sequenced in parallel at Tempus in order to ensure a properly blinded study. Subsequent to sample send out, the Tempus xO assay was run on each of the 19 samples and variants were identified using Tempus Labs' computational pipeline and algorithms. Neogenomics returned reports detecting 42 variants present within the 19 samples, 41 of which were detected by the Tempus xO assay. The discordant variant was a TP53 p.Gly112Asp at 5% variant allele fraction that was not reported by the Tempus xO assay because it is known to be benign and was therefore filtered by variant analysis.

In order to study copy number variation, amplification results were analyzed for 22 clinical samples previously identified as amplified or normal via FISH at NMH. Of these samples, six were known positives for ERBB2 (HER2) amplification and sixteen were known negatives. When analyzed by the Tempus xO pipeline, all six positives samples were identified as amplified for ERBB2 (6/6) and all 16 negatives were correctly identified as negatives. Furthermore, three replicates each of 6 cell lines (18 samples) were analyzed via the xO assay for known amplifications or losses previously reported in the literature (Supplementary Table 5). Supplementary Table 5 shows the sixteen gains/losses selected prior to analysis, and all sixteen were detected following CNV calling by the xO bioinformatics pipeline. When analyzing xO assay performance in aggregate, 64 variants were analyzed, of which 63 were detected, resulting in a 98.4% sensitivity for the Tempus xO DNA sequencing assay.

### Assay specificity

Subsequent to sensitivity analysis, Tier 1 clinically actionable xO assay genes were assessed to estimate the specificity of the assay. This study was conducted with the MCTP laboratory due to the wider array of targets present in the Michigan Oncoseq panel compared to the Neogenomics panel. A total of 33 reportable Tier 1 variants were identified from 22 samples tested at Tempus Labs and MCTP. Of these 33 variants, 32 were determined to be concordant by comparison against MCTP data. The one variant determined not to be concordant was an ATRX SNP. This variant was well covered in the Tempus sample and present at low VAF, but not present in the MCTP VCF. While this variant was not determined to be concordant with MCTP, the sample was re-sequenced by ACGT, Inc., which confirmed the presence of the ATRX variant in the sample. As such, Tempus identified zero false positives from the xO assay over the tier 1 gene targets as determined via secondary confirmation. Similarly, there were 113 variants called by Tempus in the tier 2 gene list. Of these, 107 were confirmed to be true positives when compared against previous clinical data. This resulted in a tier 2 assay positive predictive value of 94.7%.

To analyze copy number alterations for specificity, we examined 16 samples tested by FISH that were known to be negative for HER2 amplification. Within these 16 samples, there were no data indicating amplification that would rise to the threshold specified in the xO assay. As such, all 16 samples were identified as HER2 normal/equivocal.

In aggregate, there were 49 variants assessed for specificity with zero false positives. Therefore, overall specificity of the assay for SNVs, indels and CNVs was >99.9%.

### Analytic sensitivity and specificity of gene fusions

In order to analyze the sensitivity of the fusion assay, a set of consensus fusions were selected from the literature for a set of commonly available cancer cell lines as described in methods. Consensus fusions were detected at 92% sensitivity. Those fusions contained in our clinically-actionable tier (Supplementary Table 3) were detected with 100% sensitivity. The clinically-actionable MCF-7 cell line ESR1-CCDC170 fusion was detected in all 12 replicate samples. The positive predicted value for the synthetic dataset is 1.0 for the p.arc algorithm. The positive predictive value in the spike-in reference dataset is 0.95 (0.92 for the ensemble).

## DISCUSSION

For a cancer patient today, there are a wide array of potential diagnostic tests and intervention strategies. One clear path that has emerged is sequencing of tumor and germline DNA to accurately determine somatic tumor mutations, then targeting the actionable mutations. For many indications, targeted therapy based on tumor genetics or genomics is already standard of care (NCCN: Melanoma, Colorectal, Lung). However, with growing frequency, there are driver mutations that can be targeted with off-label drug indications [7, 8, 9]. Also growing are the numbers of clinical trials that involve the testing of panels of genes to qualify a patient for enrollment [10, 11]. As a result, genome informed therapeutic options have proliferated and ever more patients are considered for treatments based on NGS assays. Recent studies have indicated that clinical care is guided by NGS assay results for 30-40% of patients receiving such testing [12, 13, 14, 15, 16]. Advances in immunotherapies are also driving the need for testing genomic status of patients' tumors. For example, the FDA for the first time approved a targeted cancer drug in a tissue agnostic fashion, whereby the PD-1 inhibitor pembrolizumab was approved for microsatellite instability-high (MSI-H) or mismatch repair deficient (dMMR) solid tumors regardless of the tissue of origin [17].

We have developed an NGS-based diagnostic test to accurately detect clinically relevant genomic alterations across 1,711 cancer genes plus genome wide unbiased fusion detection in routinely processed FFPE clinical specimens along with blood or saliva for germline. This assay is unique in its use of matched tumor and normal DNA plus RNA-seq to give a comprehensive view of actionable alterations for cancer therapy and clinical trial enrollment. Through the creation of pooled cell-line models spanning key determinants of detection accuracy for somatic alterations - including MAF, indel length, degree of stromal admixture and amplitude of copy number change - we validated this test. And by examining concordance in tumors clinically characterized for selected mutations by validated tests in three different reference laboratories, we were able to corroborate accuracy in FFPE tissue specimens (see Supplementary Table 6 for summary statistics).

Because optimized large panel NGS has the ability to reveal a much wider range of genomic alterations than single gene or hotspot assays, especially when working with limited tissue samples, NGS-based genomic profiling can be used to take full advantage of targeted therapy options. Additionally, broad based NGS genomic profiling enables patients with uncommon tumors or rare genomic alterations to be identified for clinical trials. The Tempus xO assay provides the opportunity to screen known actionable gene variants, as well as a broad set of biologically relevant cancer related genes on a clinically validated platform.

## MATERIALS AND METHODS

### Assay design overview

The Tempus xO assay combines a 1,711 gene targeted tumor and normal DNA sequencing panel with tumor RNA sequencing to detect somatic and germline variants, as well as fusion mRNAs created from chromosomal rearrangements. This assay is capable of detecting somatic and germline single nucleotide polymorphisms (SNPs), indels, copy number variants, and gene rearrangements causing chimeric mRNA transcript expression. The assay is designed to identify actionable oncologic variants in a wide array of solid tumor types. It makes use of FFPE tumor samples and matched normal blood or saliva samples. The subtraction of variants detected in the normal sample from variants detected in the tumor sample gives greater somatic variant calling accuracy. Incidental pathogenic germline findings are reported per American College of Medical Genetics (ACMG) guidelines [18]. This targeted gene panel has been divided into a clinically actionable tier, wherein 130 tier 1 genes (Supplementary Table 2) that can influence treatment decisions are assayed with an assigned detection cutoff of 5% variant allele fraction (VAF) i.e. the limit of detection is 5% VAF or lower, and a secondary tier, wherein an additional 1,581 genes are assayed for analytical purposes with an assigned detection cutoff of 10% VAF (limit of detection 10% VAF or lower). The RNA based gene rearrangement detection is also divided into a primary clinically-actionable tier containing 40 rearrangements (Supplementary Table 3), and a secondary tier that contains all known fusions within the wider literature or novel fusions of putative clinical importance detected by the assay.

### Laboratory methods

#### Nucleic acid extraction

Germline DNA was extracted from either 650 ul of saliva collected in an Oragene sample tube or 400 ul of blood collected in a PAXGene blood tube. Oragene and PAXGene tubes were selected because they contain preservatives to stabilize the samples and thus mitigate issues related to transportation time and/or temperature fluctuations. Tumor total nucleic acid was extracted from formalin-fixed paraffin-embedded (FFPE) tumor tissue sections that were macrodissected (if needed based on Pathologist assessment of tumor cellularity) and proteinase K digested. For all sample types, total nucleic acid was extracted with a Chemagic360 instrument using a source-specific magnetic bead protocol and stored at 4°C if less than 24 hours and −80°C if longer. Total nucleic acid was utilized for all DNA library construction. RNA was purified from the total nucleic acid by DNaseI digestion and magnetic bead purification. Nucleic acids were quantified by a Quant-iT picogreen dsDNA reagent Kit or Quant-iT Ribogreen RNA Kit (Life Technologies), and quality was confirmed using a LabChip GX Touch HT Genomic DNA Reagent Kit or LabChip RNA High HT Pico Sensitivity Reagent Kit (PerkinElmer).

#### DNA library construction

One hundred nanograms of DNA for each tumor and normal sample was mechanically sheared to an average size of 200 base pairs using a Covaris ultrasonicator. The libraries were prepared using the KAPA Hyper Prep Kit. Briefly, DNA undergoes enzymatic end-repair and A-tailing, followed by adapter ligation, bead-based size selection, and PCR. After library preparation, each sample was hybridized to a custom designed probe set. Recovery and washing of captured targets was performed using the SeqCap hybridization and wash kit. The captured DNA targets were amplified using the KAPA HiFi HotStart ReadyMix. The amplified target-captured libraries were sequenced to a minimum unique on target depth of 500X on an Illumina HiSeq 4000 System utilizing patterned flow cell technology. Samples were further assessed for uniformity with each sample required to have 95% all targeted base pairs within the panel sequenced to a minimum depth of 300x (Supplementary Figure 1). This was determined to be sufficient for high sensitivity variant calling and copy number analysis. During analysis, any variant candidate under interrogation was flagged if the locus was determined to be insufficiently deep. These were then reviewed when present in a targeted exon. Samples shown to have insufficient depth of coverage were flagged for quality review or failed during the clinical analysis process.

#### RNA library construction

One hundred nanograms of RNA per tumor sample was fragmented with heat in the presence of magnesium to an average size of 200 base pairs. The RNA then underwent first strand cDNA synthesis using random primers, followed by combined second strand synthesis and A-tailing, adapter ligation, bead-based cleanup, and library amplification. After library preparation, samples were hybridized with the IDT xGEN Exome Research Panel. Target recovery was performed using Streptavidin-coated beads, followed by amplification using the KAPA HiFi Library Amplification Kit. The RNA libraries were sequenced to obtain at least 50 million reads on an Illumina HiSeq 4000 System utilizing patterned flow cell technology.

#### Biological sample descriptions

24 tumor/normal paired FFPE samples were processed and sequenced according to validation protocols to verify that the assay performed as expected with FFPE tissue. 24 total blood and 24 total saliva samples were processed and sequenced in the same manner and according to their own specific protocols to verify that the assay also performed well with germline samples. The blood samples were collected in PAXgene Blood DNA tubes (Catalog #761115) and the saliva samples were collected in Oragene DNA saliva kits (Catalog #OG-510).

#### Cell lines

Multiple well-characterized cell lines were used for validation of variant calling. These cell lines were chosen because they have been sequenced at a very high depth in the literature and also because they have tumor/normal pairs. These cell lines are HCC1143, HCC1143BL, HCC1954, HCC1954BL, NCI-H1672, NCI-HBL1672, NCI-H1770, NCIBL1770, NCI-H2107, NCIBL2107, COLO829, and COLO829BL. To recreate allelic fraction within a sample these cell lines were sequenced at 100% purity as well as mixtures of each tumor/normal pair at 75%, 50%, 25%, and 12.5% tumor. In addition to the cell lines, one HD200 sample from Horizon Discovery was run in multiple replicates because this sample has known, well characterized allelic fractions.

#### Clinical samples

Clinical germline samples submitted to the laboratory were required to be in one of two approved collection containers. For blood, this was the PAXgene Blood DNA tube (Catalog #761115) and for saliva samples, this was the Oragene DNA saliva kit (Catalog #OG-510). For FFPET tumor samples, Tempus pathologists review the slides to assess overall tumor amount and percent tumor cellularity as a ratio of tumor to benign nuclei. If macrodissection is necessary to obtain the minimum required 20% tumor, or if macrodissection could substantially improve tumor percentage without compromising tumor amount, the pathologist directs that the tissue be macrodissected by circling the area to be dissected on the slide. Following review, laboratory technologists macrodissect the FFPE slides and DNA and RNA are extracted per the *Nucleic Acid extraction* protocol above.

### *In silico* analysis of algorithm performance

In order to test algorithmic performance, a series of *in silico* titrations were made at a range of tumor purities (25%, 15%, 5%). Variants were generated from the COSMIC database and digitally inserted into normal sequencing results from healthy volunteers (herein referred to as “synthetic tumor samples”). At each stated variant fraction, the normal samples and synthetic tumor samples were analyzed with the same bioinformatics pipeline subsequently used for clinical samples.

### Variant calling and classification

All somatic variants are evaluated for clinical actionability using the 4-tiered system described by Li *et al.* [19] with variants that do not fall at known driver event sites undergoing further evaluation using the variant prioritization described by Dienstmann *et al.* [20] supplemented with a modified version of the ACMG classification rules [21]. All germline variants are assessed for pathogenicity using the ACMG rules.

### Clinical analytic methods

#### Patient sample analysis

In order to assess the xO assay's SNP and indel detection performance within a clinical workflow, 22 tumor/normal matched patient samples were analyzed at both Tempus and a CLIA/CAP accredited laboratory (Michigan Center for Translational Pathology (MCTP)) with the xO assay. Sensitivity and PPV were measured, as well as variant concordance between laboratories.

#### CNV validation

In order to assess sensitivity of CNV detection, a set of clinical and cell line samples with known amplifications were obtained for analysis. 20 clinical samples and 5 cell lines with three replicates each were analyzed. Within this sample set, there were 20 known amplifications of clinically important cancer genes. A further analysis was carried out in breast cancer samples wherein IHC or FISH was performed for ERBB2 (HER2) amplification. Limit of detection analysis was performed by titering cell lines with known amplifications at serially lower levels until the amplifications could no longer be detected. The assay validation was performed to assess detection of high level amplifications (defined as greater than or equal to 6 copies as determined by the algorithm), and homozygous deletions down to 30% tumor purity. The presence or absence of a copy number change is reported, but not exact copy number.

#### Fusion validation

In order to detect fusions, the Tempus xO assay relies on the unbiased detection of chimeric splice junctions in transcriptome capture RNA-sequencing data. This method is used in a wide variety of algorithms, and has met with significant success [22, 23]. Reads detected as spanning chimeric splice junctions or reads with discordant mates are used to generate candidate sites for further fusion analysis. For each of these sites, the supporting evidence is calculated, and if a pre-determined threshold is reached, the fusion candidate is reported by the software.

Despite being heavily-studied, cell lines such as MCF-7 and HCC1954 do not have complete or consensus sets of fusions (Supplementary Table 4). To avoid biases due to isolated results from a single study, a ‘gold standard’ set of fusions was determined using the union of a set of peer reviewed papers. 12 replicates of MCF-7 RNA-sequencing libraries were used to generate the data in order to estimate inter-run concordance. When evaluating thresholds for positivity, it was determined through these replicates that 16 aligned reads spanning the breakpoint were sufficient for consistent detection of known positive fusions while minimizing false positive detection calls. To further characterize sensitivity, known positive fusion samples obtained from Seracare and Horizon DX were sequenced and analyzed via the Tempus fusion detection assay. 16 of 16 positive control fusion mRNAs were detected from these samples.

To evaluate the PPV of the algorithm, sequencing data from synthetic RNA fusions [24] and a reference dataset generated using spiked-in RNA were utilized. The evaluated precision value in the synthetic fusion dataset is 1.0 for p.arc (p.arc is the RNA based translocation and fusion calling algorithm developed by the MCTP), and the evaluated precision value in the spike-in reference dataset is 0.95.

In order to analyze fusion detection performance over time and across instruments, the Horizon DX HD784 sample was sequenced repeatedly over a period of several months on two separate sequencers. Over 25 replicates two known positive fusions (ALK-EML4, ROS1-SLC34A2) were consistently detected.

## SUPPLEMENTARY MATERIALS FIGURES AND TABLES














