# Supplementary material for: Clinical validation of the Tempus xO assay
Source: Oncotarget. 2018 May 25;9(40):25826–32. doi: 10.18632/oncotarget.25381 (PMC5995233; doi:10.18632/oncotarget.25381)
Supplement: Supplementary file 1 [file oncotarget-09-25826-s001.pdf]

Clinical validation of the Tempus xO assay

SUPPLEMENTARY MATERIALS

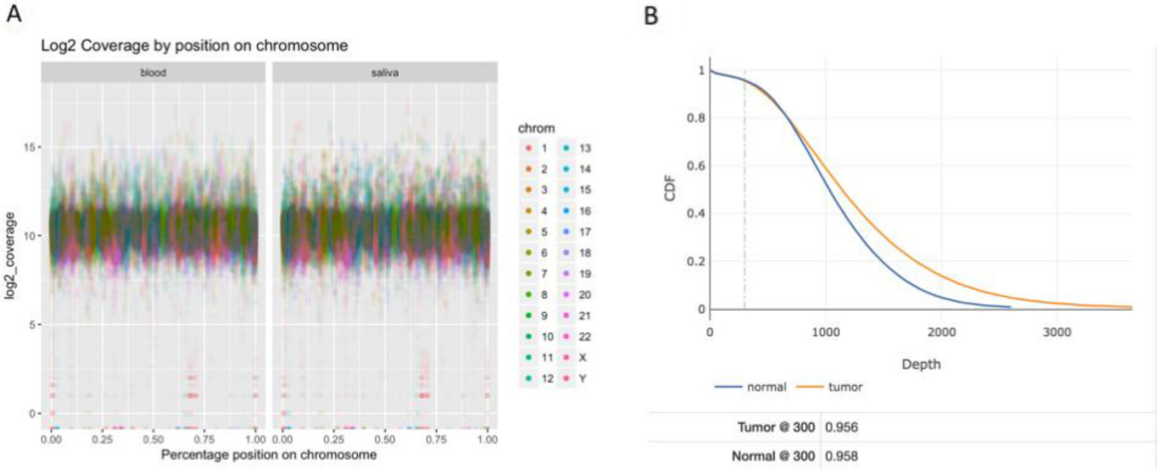

Supplementary Figure 1: Coverage uniformity across clinical samples for the xO assay derived from both blood and saliva as starting substrates. **(A)** De-duplicated coverage for 18 representative tumor-normal pairs were analyzed to determine uniformity of coverage across the xO target regions both for saliva, blood, and FFPE. Colors represent coverage across each chromosome. normalized for differential genomic length. **(B)** 1 - CDF representing percentage of base pairs covered (y-axis) greater than a specific sequencing depth (x-axis). Samples being run on the xO assay were required to have 99% of target regions greater than 100x depth and 95% of target regions covered greater than 300x depth.

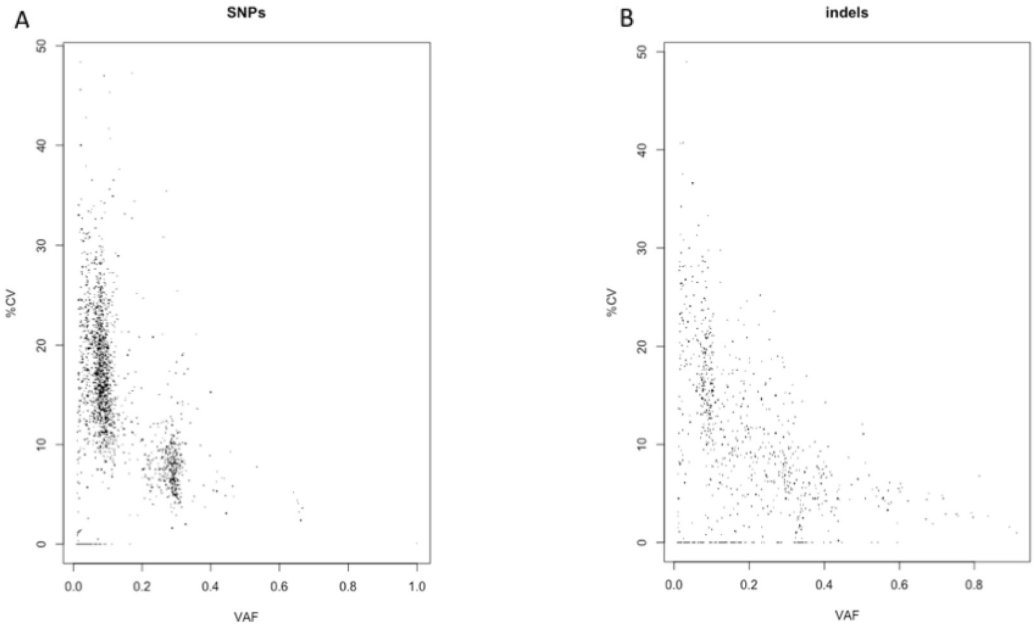

Supplementary Figure 2: Coefficient of variation (CV) for positive control variants sequenced at Tempus. **(A)** Coefficient of variation analyzed across variant allele fractions for SNP's from Horizon DX HD200 samples run over the course of 4 months encompassing 25 replicates on two sequencers. **(B)** Coefficient of variation analyzed across variant allele fractions for indels from A.

Supplementary Table 1: Tempus xO oncology panel, 1711 gene list.

See Supplementary File 1

Supplementary Table 2: Tempus xO assay Tier 1 genes.

See Supplementary File 2

Supplementary Table 3: 41 clinically-actionable RNA based gene rearrangement.

See Supplementary File 3

Supplementary Table 4: Consensus fusions.

See Supplementary File 4

Supplementary Table 5: Copy number variation loci in previously characterized cell lines.

See Supplementary File 5

Supplementary Table 6: Summary statistics.

See Supplementary File 6
