# Supplementary material for: Clinical validation of the Tempus xO assay
Source: Oncotarget. 2018 May 25;9(40):25826–32. doi: 10.18632/oncotarget.25381 (PMC5995233; doi:10.18632/oncotarget.25381)
Supplement: Supplementary file 5 [file oncotarget-09-25826-s005.docx]

**Supplementary Table 4: Consensus fusions**

**Table 4A. Literature-based Fusion calls in MCF-7**

Combined Calls: Each fusion is present in more than 1 of 4 sources(see Appendix 4.3).

| Gene_A | Gene_B | Times detected (/12) |
| --- | --- | --- |
| ARFGEF2 | SULF2 | 12 |
| BCAS4 | BCAS3 | 12 |
| RPS6KB1 | TMEM49 | 12 |
| GCN1L1 | MSI1 | 12 |
| SULF2 | PRICKLE2 | 12 |
| ATXN7L3 | FAM171A2 | 11 |
| SMARCA4 | CARM1 | 12 |
| MYO9B | FCHO1 | 12 |
| AHCYL1 | RAD51C | 12 |
| BCAS4 | ZMYND8 | 12 |
| MYH9 | EIF3D | 12 |

**Table 4B. Literature-based Fusion calls in HCC-1954**

5 Combined calls. Each is present in more than 1 of 4 sources.

| Gene_A | Gene_B | Times detected (/12) |
| --- | --- | --- |
| PHF20L1 | SAMD12 | 12 |
| STRADB | NOP58 | 11 |
| NSD1 | "" | 12 |
| PVT1 | CLPTM1L | 12 |
| UIMC1 | RAP80 | 12 |

**References:**

MCF Source1: **Detecting and visualizing gene fusions**

<http://dx.doi.org/10.1016/j.ymeth.2012.09.013>

| ARFGEF2 | SULF2 |
| --- | --- |
| BCAS4 | BCAS3 |
| RPS6KB1 | TMEM49 |
| GCN1L1 | MSI1 |
| SULF2 | PRICKLE2 |
| ATXN7L3 | FAM171A2 |
| SMARCA4 | CARM1 |
| MYO9B | FCHO1 |
| AHCYL1 | RAD51C |
| *BCAS4 | *ZMYND8 |
| *MYH9 | *EIF3D |
| ABCA5 | PPP4R1L |
| BCAS3 | ATXN7 |
| C16orf45 | ABCC1 |
| GATAD2B | NUP210L |
| NAV1 | GPR37L1 |
| USP31 | CRYL1 |
| TANC2 | CA4 |
| TAF4 | BRIP1 |
| SYTL2 | PICALM |

*: This fusion was partially validated in the source and congruent with other literature sources.

MCF Source 2: **A novel bioinformatics pipeline for identification and characterization of fusion transcripts in breast cancer and normal cell lines.**

doi:10.1093/nar/gkr362

| ARFGEF2 | SULF2 |
| --- | --- |
| BCAS4 | BCAS3 |
| GCN1L1 | MSI1 |
| SULF2 | PRICKLE2 |
| ATXN7L3 | FAM171A2 |
| MYH9 | EIF3D |
| RPS6KB1 | DIAPH3 |
| ADAMTS19 | SLC27A6 |

MCF Source 3: **Identification of fusion genes in breast cancer by paired-end RNA-sequencing**

<http://genomebiology.com/2011/12/1/R6>

| ARFGEF2 | SULF2 |
| --- | --- |
| BCAS4 | BCAS3 |
| RPS6KB1 | TMEM49 |

MCF Source 4: **Functionally recurrent rearrangements of the MAST kinase and Notch gene families in breast cancer.**

<http://dx.doi.org/10.1038/nm.2580>

| ARFGEF2 | SULF2 |
| --- | --- |
| BCAS4 | BCAS3 |
| RPS6KB1 | TMEM49 |
| SMARCA4 | CARM1 |
| MYO9B | FCHO1 |
| AHCYL1 | RAD51C |
| BCAS4 | ZMYND8 |
| PAPOLA | AK7 |
| TRIM37 | VMP1 |
| ARHGAP19 | DRG1 |
| PVT1 | MYC |

**HCC-1954 Source 1: Functionally recurrent rearrangements of the MAST kinase and Notch gene families in breast cancer**

<http://dx.doi.org/10.1038/nm.2580> (Supp.Data 2)

| *NSD1* | *""* |
| --- | --- |
| *PVT1* | *CLPTM1L* |

**HCC-1954 Source 2: Complex landscapes of somatic rearrangement in human breast cancer genomes**

[*10.1038/nature08645*](https://dx.doi.org/10.1038%2Fnature08645) *(Table 3)*

| *STRADB* | *NOP58* |
| --- | --- |

**HCC-1954 Source 3:**   **Transcriptome-guided characterization of genomic rearrangements in a breast cancer cell line** [*10.1073/pnas.0812945106*](https://dx.doi.org/10.1073%2Fpnas.0812945106) *(Table1)*

| *SAMD12* |  |
| --- | --- |
| *PDCD1LG2* | *C18orf10* |
| *MRE11A* | *""* |
| *PHF20L1* | *SAMD12* |
| *NSD1* | *""* |
| *PVT1* | *CLPTM1L* |
| *UIMC1* | *RAP80* |

**HCC-1954 Source 4**: **A novel bioinformatics pipeline for identification and characterization of fusion transcripts in breast cancer and normal cell lines.**

doi:10.1093/nar/gkr362

| PHF20L1 | SAMD12 |
| --- | --- |
| STRADB | NOP58 |
| INTS1 | PRKAR1B |
| GSDMC | PVT1 |
