# Supplementary material for: Clinical validation of the Tempus xO assay
Source: Oncotarget. 2018 May 25;9(40):25826–32. doi: 10.18632/oncotarget.25381 (PMC5995233; doi:10.18632/oncotarget.25381)
Supplement: Supplementary file 6 [file oncotarget-09-25826-s006.docx]

**Supplementary Table 5: Copy Number Variation Loci in Previously Characterized Cell lines**

| **Cell Line** | **Gene** | **Detected** |
| --- | --- | --- |
| COLO829 | BRAF | Yes |
| HCC1954 | MYC | Yes |
| NCIH2107 | MYC | Yes |
| HCC1143 | MYC | Yes |
| HCC1143 | AKT1 | Yes |
| NCIH1770 | MYCN | Yes |
| HD753 (HznDx) | MYCN | Yes |
| NCIH1770 | MET | Yes |
| HD753 (HznDx) | MET | Yes |
| NCIH2107 | MYCL | Yes |
| HD753 (HznDx) | MYCL | Yes |
| HCC1143 | CCND1 | Yes |
| HCC1954 | CCND1 | Yes |
| HCC1954 | ERBB2 | Yes |
| HCC1143 | PRBM1 | Yes |
| HCC1143 | FGF3 | Yes |

**5. Resource for public copy number variation data on cell lines:**

<http://igrcid.ibms.sinica.edu.tw/cgi-bin/index.cgi>

**6. Resource for cell line called copy number data from Foundation Medicine study:**

<http://www.nature.com/nbt/journal/v31/n11/extref/nbt.2696-S1.pdf>

**7. Characterization of COLO829 Cell lines as a reference standard**

<https://www.ncbi.nlm.nih.gov/pmc/articles/PMC4837349/>
